# Field monitoring data on a residential exhaust air heat pump system (air-to-air heat pump)

**DOI:** 10.1016/j.dib.2021.107386

**Published:** 2021-09-20

**Authors:** Arsalan Shirani, Alexander Merzkirch, Jennifer Roesler, Stephan Leyer, Frank Scholzen, Stefan Maas

**Affiliations:** a Université du Luxembourg, 6, rue Richard Coudenhove-Kalergi, Luxembourg 1359, Luxembourg; b Bosch Thermotechnology, Junkersstraße 20, (Neckar), Wernau 73249, Germany

**Keywords:** Exhaust air heat pump, Ventilation based heating system, Heat recovery ventilation, Efficient residential buildings, EHA1, Exhaust air condition 1 (After air-to-air heat exchanger), EHA2, Exhaust air condition 2 (After evaporator), ETA, extract air from building, Evap., Evaporator, COP, Coefficient of performance, HEX, heat exchanger, HP, heat pump, HRV, Heat Recovery Ventilation, ODA, Outdoor Air, SUP1, Supply Air condition 1 (after air-to-air heat exchanger), SUP2, Supply Air condition 2 (after condenser), T, Temperature, X, Humidity

## Abstract

This data article presents the raw data used in the article “Experimental and analytical evaluation of exhaust air heat pumps in ventilation-based heating systems” [1]. The data set contains measurement results of a field monitoring on a residential exhaust air heat pump system (air-to-air heat pump) in Germany. This data could be used to investigate the dynamic behavior and performance of the exhaust air heat pump systems. The data set contains air temperature and humidity of all four sides of the heat pump unit. Moreover, the electrical consumption of the unit and the dynamic pressure difference on the exhaust side (as indication of the air volume rate) could be also found in the data set.

## Specifications Table

**Table:** 

Subject	Renewable Energy, Sustainability and the Environment
Specific subject area	Air to Air Heat Pump Technology
Type of data	MATLAB Data Figure
How data were acquired	Type K Thermocouple for temperature STPH-2–1–05 for humidity Pitot tube for dynamic pressure difference Testo 6351 for pressure difference transmitter Current transformer for electrical power Tracer gas for air volume rate
Data format	Raw and smoothed
Parameters for data collection	Humidity data was recorded as relative humidity and converted to absolute humidity. Dynamic pressure difference was used as an indication of air volume rate.
Description of data collection	The measurement period was 55 winter days with an interval of 1 second. Logging and conversion of signals were conducted using a modular I/O system; the fieldbus controller Ethernet 750–881 from WAGO Kontakttechnik.
Data source location	Stuttgart, Germany
Data accessibility	Descriptions and figures are provided with the article, measurement data is uploaded in a public repository repository name: Mendeley Data Shirani, Arsalan (2021), “Field monitoring data on a residential exhaust air heat pump system (air to air heat pump)”, Mendeley Data, v3 [2] Link to raw date: http://dx.doi.org/10.17632/smymkjcbxx.3
Related research article	Authors’ names Arsalan Shirani Alexander Merzkirch Jennifer Roesler Stephan Leyer Frank Scholzen Stefan Maas Title Experimental and analytical evaluation of exhaust air heat pumps in ventilation-based heating systems Journal Journal of Building Engineering https://doi.org/10.1016/j.jobe.2021.102638

## Value of the Data

• The presented data could be used for better understanding the functionality of exhaust air to air heat pump and heat recovery ventilation technologies and compare the heat pump power and performance to the other heat pump technologies.
• The data is interesting for the building energy engineers, heating system developers and researchers working on the heat pump and heat recovery technologies.
• The data could be used to understand and model the dynamic behavior of exhaust air heat pumps and heat recovery ventilation units.

## 1 Data Description

The data set containing two MATLAB structures is uploaded in a public repository [2]. Each field of the MATLAB structures contains one raw measured data or one smoothed measured data. Data smoothing is conducted using MATLAB function “*smoothdata*” with a Gaussian-weighted moving average filter with a window length of 120 time steps. Raw and smoothed measured data of the air temperature [in°C] and air humidity [in kg/kg] on all four side of the unit could be found on the data set [2]. Moreover, the measured electrical consumption of the unit [in Watt], as well as dynamic pressure difference on the exhaust air side of the unit [in Pa] are available on the data set. The date and time of the measured data is also available in “date” field. Measurement period was 55 winter days (during December 18th, 2019 and February 12th, 2020) with an interval of 1 second.

## 2 Experimental Design, Materials and Methods

**Fig. 1:**
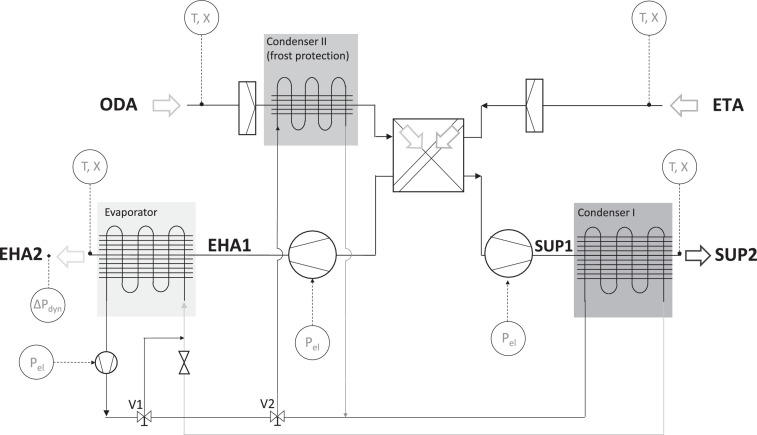
Simplified illustration of the measured EHA HP [1].

Fig. 1 shows a hydraulic illustration of the measured exhaust air heat pump (EHA-HP) and the position of the installed sensors. Air temperature and humidity on all four sides of the unit (outdoor air, extract air coming from the building, supply air to the building and exhaust air leaving the house) and electrical power consumption of the unit (compressor and ventilators) were measured and logged. Logging and conversion of signals were conducted using a modular I/O system; the Fieldbus controller Ethernet 750–881 from WAGO Kontakttechnik.

The air temperature (T) is measured with type K thermocouples from RS PRO. The calibration of thermocouples is conducted based on a calibration bath method. The air humidity (X) is measured using STHP-2–1–05 sensors from NodOn. Electrical power consumption (P_el_) of the unit is measured using plug-in current transformer from WAGO Kontakttechnik.

Table 1 gives an overview of the applied measurement methods and used sensors.

**Table 1:** Overview of applied measurement methods and sensors.

Value	Sensor/measurement method	Measurement accuracy	Manufacturer
Temperature	Type K Thermocouple	±0.004 × |t| [ °C]	RS PRO
Humidity	STPH–2–1–05	±2 [%]	NodOn
Dynamic pressure difference	Pitot tube	±2 [%]	Mueller Messinstrumente
Pressure difference transmitter	Testo 6351	±0.3 [Pa]	Testo
Power	Current transformer	±1 [%]	WAGO Kontakttechnik
Air volume rate	Tracer gas	±5 [%]	LumaSense Technologies

**Fig. 2:**
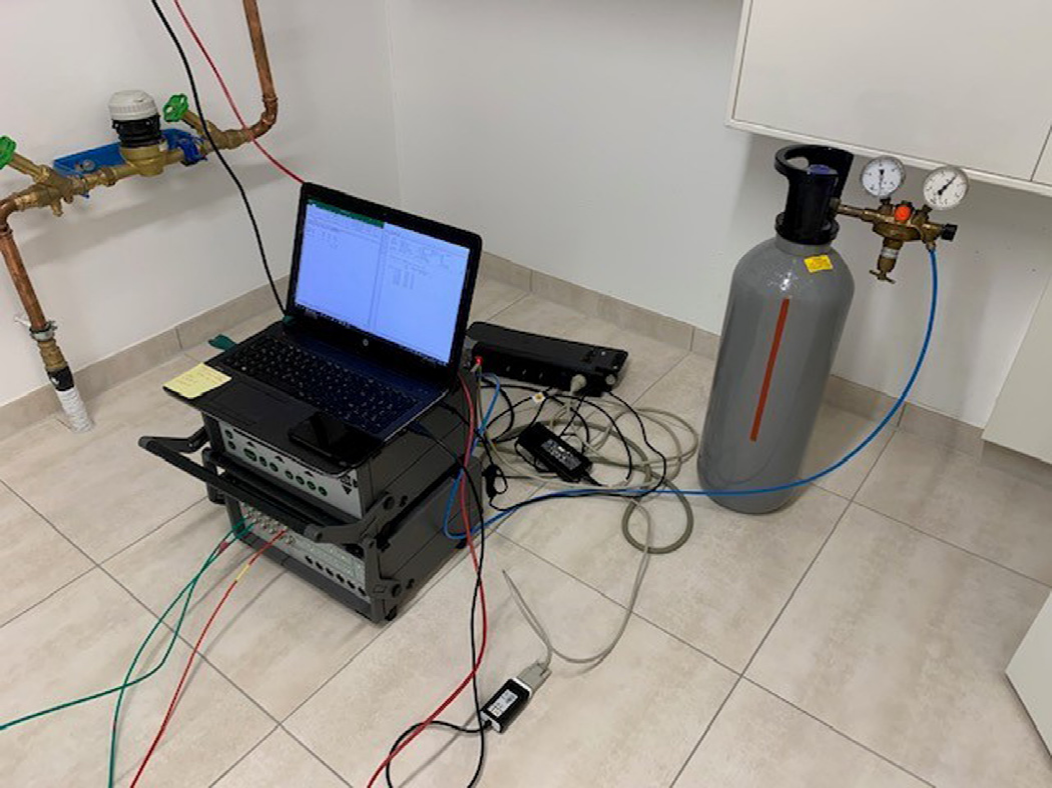
Experimental set up, tracer gas method.

In addition, the dynamic pressure difference (as an indication of the air volume rate) on the exhaust side was logged during the measurement period ($\Delta P_{\text{dyn}}$). Finally, the air volume rates of the unit at different ventilation levels was measured using Constant injection tracer gas method, as described in [3]. Fig. 2 shows the experimental set up for the tracer gas measurement.

Table 2 summarizes the measured air volume rates and ventilators’ powers usage in different levels.

**Table 2:** Summary of measurements on air volume rates.

Ventilation level	Dynamic pressure difference[Pa]	Air volume rates[m³/h]	Ventilators’ power usage [W]
1	1.5	91.2	20
2	5	184.6	50
3	12	249.4	123
4	26	341.1	334

**Fig. 3:**
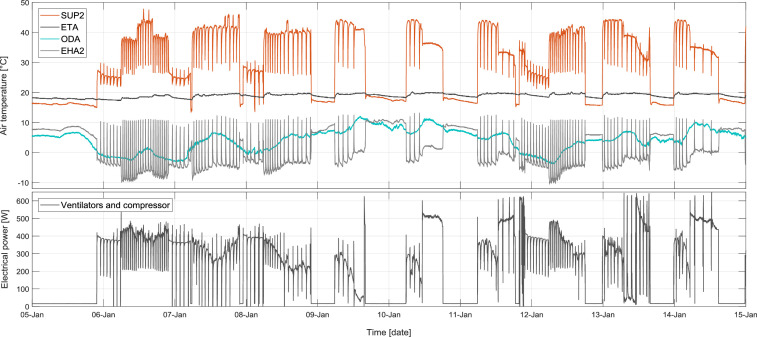
Air temperatures and electrical consumption of the unit on 10 sample winter days.

The measurements were conducted in an efficient house with a living area of 185 m² in Stuttgart, Germany. The EHA-HP was installed in a ventilation-based hybrid heating system as the central heating source of the building. Fig. 3 illustrates the air temperatures and the electrical consumption of the measured exhaust air heat pump on 10 sample days during the measurements period.

**Fig. 4:**
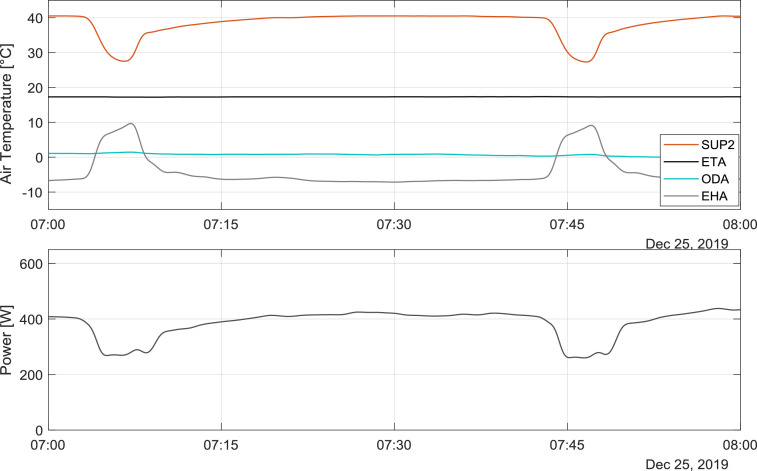
Sample of a defrost process.

Every drop in the SUP2 temperature shows a defrosting process of the heat pump's evaporator. During every defrosting process the evaporator is heated up using heated refrigerant via the provided bypass valve (V1) in the refrigerant circuit (see Fig. 1), for a duration of around five minutes. The process leads to a temperature rise in the EHA2 temperature (exhaust air leaving the unit). Fig. 4 shows a sample of two defrost processes in one hour. It could be seen that in this process the evaporator would be heated up and the condenser is discharged, that is why the EHA temperature increases and the SUP2 temperature reduces.

Table 3 gives an overview of the measured electrical energy consumption as well as minimum and maximum power of the power consumption of the unit. Moreover, based on number of defrost cycles and heat pump run time, it could be seen that the heat pump runs a defrost process approximately every one hour.

**Table 3:** Summary of measured data of the exhaust air heat pump.

Description	Value	Unit
Total electrical energy input	261	[kWh]
Minimum power (HRV mode, ventilators level 1)	20	[W]
Maximum power (heating mode, ventilators level 2)	650	[W]
Number of defrost cycles	667	[Cycles]
Heat pump run time	681	[Hours]

**Fig. 5:**
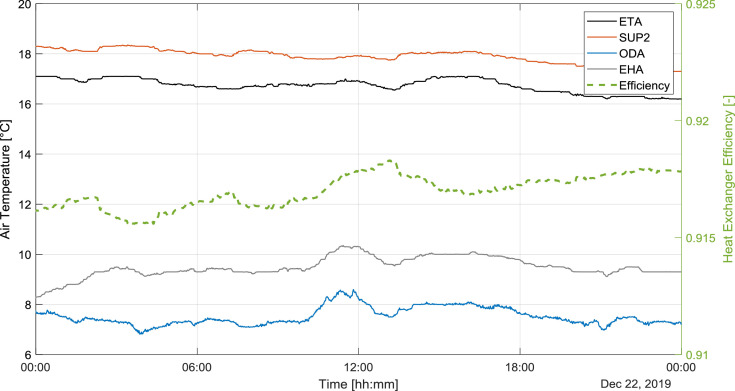
Air temperatures in the heat recovery ventilation mode.

The periods, which the SUP2 (temperature after the unit) is lower than the ETA (extract air temperature coming from building), the unit works in heat recovery ventilation mode and the heat pump is switched off. In this mode, the electrical consumption of the unit is around 50 W and it contains the electrical consumption of the ventilators. Fig. 5 shows a sample day, in which the unit works only in the heat recovery ventilation mode. The heat recovery efficiency (η) is calculated using the following equation:

$$\eta =\;\frac{\vartheta_{SUP}-\;\vartheta_{ODA}}{\vartheta_{ETA}-\;\vartheta_{ODA}}$$

Table 4 summarizes the measured air temperatures of the unit. The average values of SUP2 and EHA2 is calculated separately for both HP run time and the whole measurement period.

**Table 4:** Summary of measured temperatures, including min, max and average values.

Description	Value	Unit
Average outside air temperature (ODA)	4.1	[°C]
Average extract air temperature (ETA)	18.5	[°C]
Average supply air temperature (SUP2)	20.9	[°C]
Average supply air temperature during HP run time (SUP2)	34.8	[°C]
Minimum supply air temperature (SUP2)	11.6	[°C]
Maximum supply air temperature (SUP2)	46.8	[°C]
Average exhaust air temperature (EHA2)	2.2	[°C]
Average exhaust air temperature during HP run time (EHA2)	−3.44	[°C]
Minimum exhaust air temperature (EHA2)	−11.7	[°C]
Maximum exhaust air temperature (EHA2)	16.8	[°C]

## CRediT authorship contribution statement

**Arsalan Shirani:** Conceptualization, Methodology, Software, Investigation, Data curation, Writing – review & editing, Visualization. **Alexander Merzkirch:** Conceptualization, Methodology, Investigation. **Jennifer Roesler:** Investigation. **Stephan Leyer:** Conceptualization, Supervision. **Frank Scholzen:** Conceptualization, Supervision. **Stefan Maas:** Conceptualization, Supervision.

## Declaration of Competing Interest

The authors declare that they have no known competing financial interests or personal relationships, which have, or could be perceived to have, influenced the work reported in this article.
